# Insights into imaging of aortitis

**DOI:** 10.1007/s13244-012-0192-x

**Published:** 2012-09-20

**Authors:** Diana E. Litmanovich, Afra Yıldırım, Alexander A. Bankier

**Affiliations:** 1 Department of Radiology, Beth Israel Deaconess Medical Center, Harvard Medical School, Boston, MA USA; 2 Department of Radiology, Erciyes University School of Medicine, Kayseri, Turkey; 3 Department of Radiology, Beth Israel Deaconess Medical Center, 330 Brookline Avenue, Boston, MA 02215 USA

**Keywords:** Aortitis, Vasculitis, MDCT, MR, PET-CT

## Abstract

**Background:**

Aortitis is a subtype of the more general term “vasculitis”, an inflammatory condition of infectious or noninfectious origin involving the vessel wall. The term “vasculitis” refers to a broad spectrum of diseases with different aetiologies, pathophysiologies, clinical presentations and prognoses. The clinical manifestations are nonspecific, as are the laboratory findings such as pain, fever, weight loss, vascular insufficiency and elevated levels of acute phase reactants, as well as other systemic manifestations, and sometimes may mimic other entities. Thus, if not suspected as part of the initial differential diagnosis, aortitis can be overlooked during the workup of patients with constitutional symptoms and systemic disorders. Methods: Imaging is rarely used for the primary diagnosis, but imaging findings, although nonspecific, can help in the assessment of these patients and is often required for making the final diagnosis. Imaging can be critical in the initiation of appropriate management and therapy. Results: Noninvasive cross-sectional imaging modalities such as contrast-enhanced CT, magnetic resonance (MR) imaging, nuclear medicine and in particular positron emission tomography (PET) are the leading modalities in modern diagnostic imaging of aortitis for both the initial diagnosis and follow-up. Conclusion: This review focusses on the most common manifestations of aortitis with which radiologists should be familiar.

***Teaching Points*:**

• *Aortitis is an inflammatory condition of infectious/noninfectious origin involving the vessel wall*.

• *Imaging findings can help in the assessment of aortitis and are often crucial for the final diagnosis*.

• *Contrast-enhanced CT, MRI and PET-CT are used for both the initial diagnosis and follow-up of aortitis*.

## Introduction

Vasculitis refers to a varied group of rare disorders that all share underlying inflammation of the vasculature [1, 2]. The inflammation can affect blood vessels of any size, both arteries and veins, in any location of the body [1]. The inflammation may be focal, affecting a single anatomical level within a vessel, or it may be widespread, with areas of inflammation scattered throughout the entire vascular bed of a particular organ, or may even affect more than one organ system [3]. The inflammation may be infectious or noninfectious, the latter being more common [1]. In the thorax, noninfectious inflammatory vasculitis can affect large vessels such as the aorta and the pulmonary arteries, with diseases such as Takayasu arteritis, giant cell arteritis (GCA) or Behçet's disease [4]. Medium and small vessels can be involved in the context of collagen vascular disorders such as rheumatoid arthritis, ankylosing spondylitis, relapsing polychondritis or systemic lupus erythematosus [5]. Infectious involvement caused by tuberculosis, syphilis or Salmonella is possible but less common unless pre-existing aortic wall damage is present [6]. The main aetiologies for vasculitis in the thorax are summarised in Table 1.

**Table 1:** Classification of aortitis

Types of aortitis	
Noninfectious aortitis	Infectious aortitis
Most common aetiologies	Most common aetiologies
Takayasu arteritis	Pyogenic infection
Giant cell arteritis	Tuberculous aortitis
Behçet's disease	Syphilitic aortitis
Ankylosing spondylitis	
Relapsing polychondritis	
Rheumatoid arthritis	
Idiopathic isolated aortitis	

Although imaging is seldom used for primary diagnosis of thoracic large vessel vasculitis, it plays an important role in differentiating between infectious and noninfectious vasculitis, as well as monitoring disease activity or guiding biopsy. Another important role of imaging is to exclude alternative diagnoses for the clinical presentation of patients [1, 5, 7].

The most commonly used imaging modalities in the workup of thoracic large vessel vasculitis are CT, MRI and ultrasound, including transoesophageal echo [7]. There is also a growing role for positron emission tomography [5]. The aim of this article is to present both the techniques used to assess thoracic large vessel vasculitis and to detail the particular findings characteristic for those diseases.

## Imaging techniques

Although no single specific examination protocol or algorithm exists for the assessment of potential vasculitis, the main purpose of imaging is to assess vessel wall and vessel lumen morphology using similar protocols for initial assessment and follow-up for adequate comparison. Note that the protocols used for CT or MRI are similar to those used for evaluation of acute aortic syndrome or pulmonary embolism [7].

It must, however, be emphasised that imaging is only one of many other tools available for the diagnosis of vasculitis, such as gene analysis, family studies, clinical assessment, biopsy, histology and immunochemistry [1].

### CT technique

CT is used for the assessment of aortic wall thickness and regularity, aortic diameter, mural calcifications and aortic branches. Bi-phasic CT is typically performed, including non-enhanced CT of the chest (40 mA, 100 kVp) [8]. The non-enhanced scan is used to exclude intramural haematoma and to show intramural calcification, particularly in cases of long-standing vasculitis. Therefore, it can be omitted in patients younger than 40 years of age. A contrast-enhanced scan of both the aorta and pulmonary arteries is typically the second step in the aortic assessment. Automatic exposure modulation controlling the tube current with an mA range of 200–500 and kilovoltage setting of 100 kVp is recommended for adequate assessment of the thoracic aorta [9]. To enhance the aorta and pulmonary arteries simultaneously, triggering at the level of left atrium is recommended with an injection rate of not less than 4 ml/s, and an overall injection of at least 100 ml of IV contrast is required [8]. ECG-gated CTA can be considered for better imaging of the ascending aorta [10]. Transverse images in conjunction with coronal, sagittal and curved reformats are helpful for assessment of the extent and severity of vessel wall involvement. CT angiography can also be used as a follow-up tool to assess the treatment response and/or activity of the disease [5].

### MRI technique

Similar to CT, MRI provides excellent assessment of the vascular wall and lumen with multiplanar and three-dimensional reformations, with the advantage of using no ionising radiation. This is the modality of choice for follow-up, particularly in young patients. Even when gadolinium cannot be administered because of impaired renal function, assessment of the vessel can be done with MRA techniques. A more detailed description of the findings and technique will be given while discussing specific aortitis entities.

### Nuclear imaging

18-FDG-PET-CT is playing an increasing role in assessing inflammatory changes such as vasculitis, atherosclerosis and acute dissection in the aorta and pulmonary arteries [1, 11]. As opposed to FDG PET assessment of cardiac disease where adjustment of the protocol is required because of radiotracer accumulation in normal myocardium, FDG does not accumulate in normal vascular structures. Thus, any uptake of 18-FDG in the aortic wall is abnormal because of inflammatory or infectious processes [11], and no specific protocol is needed for assessing vasculitis with PET-CT [11]. Although inflammatory activity is well appreciated on images, morphologic assessment is limited because of the relatively low special resolution; thus, nuclear imaging studies receive substantial benefit when obtained in conjunction with either CT or MRI, increasing the sensitivity and specificity of this test [12–14].

## Noninfectious aortitis

As previousy stated, vascular inflammation in aortitis is predominantly noninfectious. The vast majority of noninfectious aortitis cases are associated with rheumatic disease such as Takayasu arteritis, GCA, long-standing ankylosing spondylitis, Cogan syndrome (interstitial keratitis, iritis, conjunctival or subconjunctival haemorrhage, fever, aortic insufficiency) or relapsing polychondritis, with aortitis seen in more than 10 % of cases (Table 2) [15]. There is also a less frequent but well-documented association between aortitis and rheumatologic problems present in rheumatoid arthritis, seronegative spondyloarthropathies, Behçet's disease and SLE [5]. Sarcoidosis, Wegener's granulomatosis, polyarteritis nodosa and juvenile rheumatoid arthritis have also been associated with sporadic reports of aortic involvement [5]. Symptoms of polymyalgia rheumatica have been reported in approximately 10 % of patients with noninfectious ascending aortitis [16].

**Table 2:** Rheumatoid diseases associated with aortitis

Takayasu arteritis
Giant cell arteritis
Long-standing ankylosing spondylitis
Rheumatoid arthritis
Behçet's disease (more frequent in the Mediterranean region)
Seronegative spondyloarthropathies
SLE
Sarcoidosis
Wegener's granulomatosis
Polyarteritis nodosa, juvenile rheumatoid arthritis, Cogan syndrome—rare

### Takayasu arteritis

Takayasu arteritis (TA) is a necrotising and obliterative segmental, large-vessel panarteritis of unknown cause, involving elastic arteries including the aorta and its branches. T-cell-mediated panarteritis starts in the adventitial vasa vasorum and progresses inwards, with the unknown antigen triggering monoclonal T-cell expansion [1, 17]. This inflammatory process begins with perivascular cuffing of the vasa vasorum in the early stage of the disease followed by fibrosis and calcifications [18]. Destruction and fibrosis coexist with the former, causing aneurysmal formation and the latter leading to narrowing of the aorta and its branches, resulting in significant stenosis. Takayasu artertitis is also known as “pulseless disease” from the frequent involvement of subclavian arteries with substantial stenosis and subsequently diminished peripheral pulses [5].

This idiopathic process has a strong female predilection, affecting females ten times more than men [19]. The peak incidence is in the 3rd decade of life, but age spans from late childhood to the 5th decade have been reported [19]. The overall rate in the US is 2.6 cases per 1 million persons, with moderate Asian over-representation [1]. Two specific disease distributions have been reported, Japanese and Indian. In the Japanese group, the predilection is toward the thoracic aorta and its branches, as opposed to the Indian group, where the abdominal aorta and renal arteries are most affected [3].

#### Clinical presentation

Two phases, early acute and late chronic, are seen in TA. In the early phase, so-called “B” symptoms, such as weight loss, fatigue, night sweats, anorexia and malaise, are common [17]. Chronic phase symptoms are determined by the organs involved, with fewer constitutional symptoms, and with claudication, cerebrovascular insufficiency, carotid artery pain and renal artery involvement most frequently reported [17]. Hypertension due to renal artery involvement is particularly frequent among the Indian population [20].

Aortic aneurysm or stenosis has been reported in up to one-third of the cases [21]. In the order of frequency, aneurysmal formation is seen in descending, abdominal and ascending aortic segments. Stenosis of the aorta is even more frequent, occurring in 53 % of cases based on a National Institutes of Health series, mostly affecting the abdominal aorta (up to 70 % of aortic stenosis cases) [1].

#### Diagnosis

Takayasu arteritis is diagnosed based on the 1990 American College of Rheumatology criteria [22] (Table 2).

1. Age of onset younger than 40 years
2. Intermittent claudication
3. Diminished brachial artery pulse
4. Subclavian artery or aortic bruit
5. Blood pressure difference greater than 10 mmHg (arms)
6. Angiographic (CT, MR) evidence of stenosis

If three out of six criteria are present, the sensitivity and specificity for the diagnosis according to these criteria are 90 % and 98 % respectively. Laboratory tests such as erythrocyte sedimentation rate and C-reactive protein are elevated in 70 % of patients in the acute and 50 % of patients in the chronic stage of the disease [1].

#### Imaging findings

All clinically available imaging modalities play an important role in the diagnosis and follow-up of TA. Until recently, digital subtraction angiography (DSA) was the procedure of choice, but this has been subsequently replaced by cross-sectional imaging [23] as the major disadvantages of DSA are the high radiation dose, substantial contrast material burden and difficulty in assessing cases of long-segment stenosis and aortic wall abnormalities such as arterial calcification, wall inflammation or chronic fibrosis [5]. Additionally, transoesophageal echocardiography and intravascular US are tools that provide high-resolution images of subtle changes in aortic segments that may appear normal with other imaging techniques, although usually only the proximal aorta can be assessed [18].

**Fig. 1:**
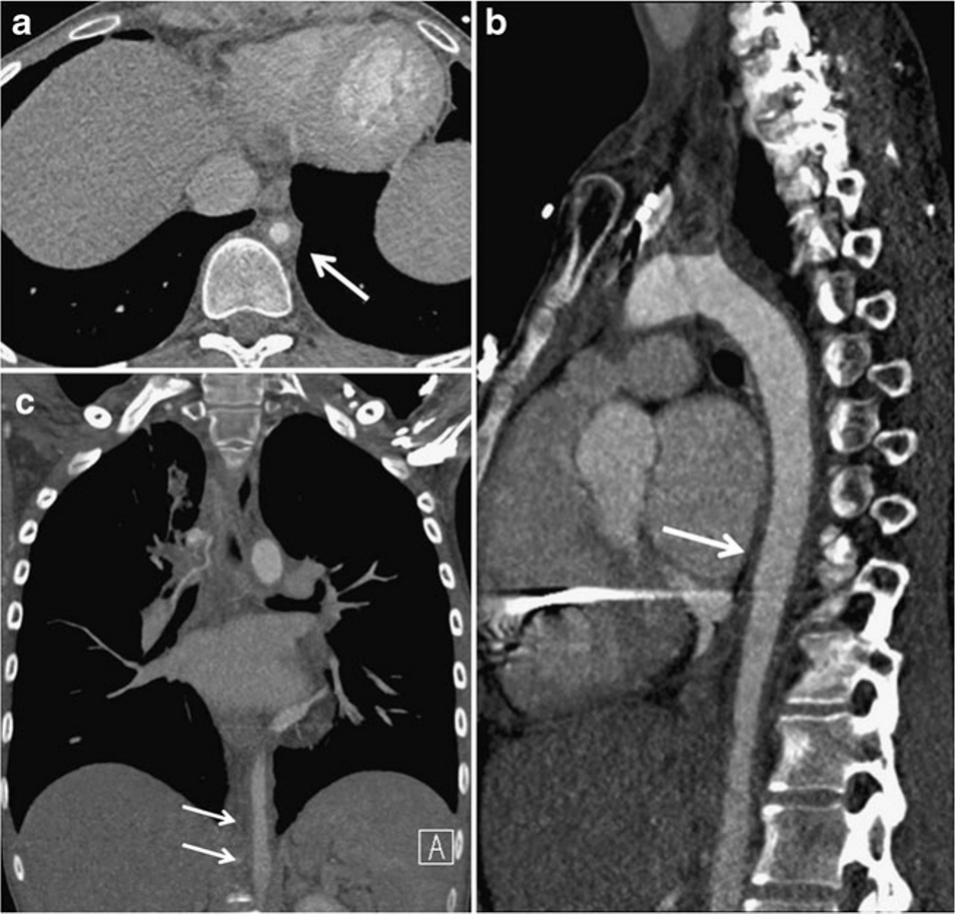
**a**–**c** A 25-year-old previously healthy man with chest and abdominal pain, weight loss and low grade fever. Axial (**a**), sagittal (**b**) and coronal (**b**) images of CTA of the torso demonstrate circumferential wall thickening of both the ascending and descending thoracic aorta as well as aortic wall thickening including in the abdominal aorta (*white arrows*). Diffuse narrowing of the aorta can be appreciated on all three views. Courtesy of Dr. Eduard Ghersin, Jackson Memorial Hospital, Miami, Florida

**Fig. 2:**
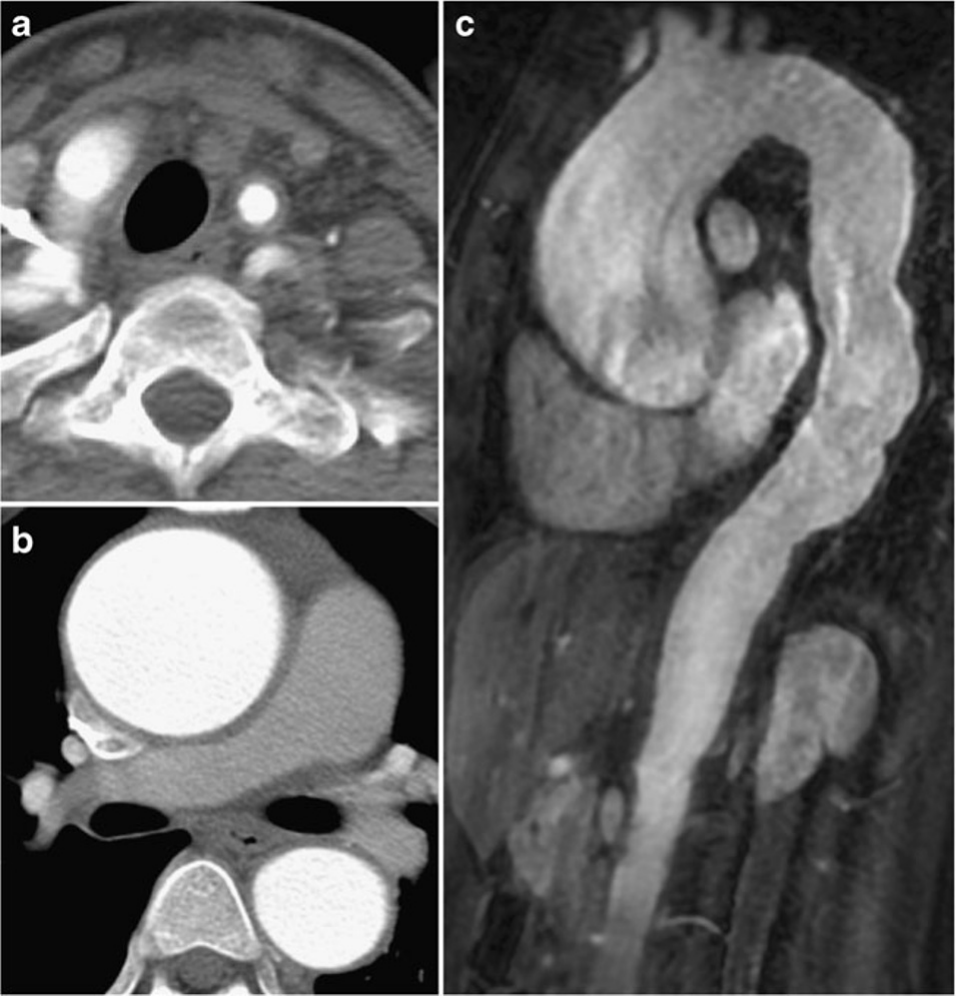
**a**–**c** 20-year-old previously healthy woman with chest pain. CTA of the lower neck demonstrates circumferential wall thickening of the left carotid artery (**a**). Ascending aorta is dilated with circumferential wall thickening (**b**). Gadolinium-enhanced sagittal (**c**) view demonstrated dilatation of both ascending and descending thoracic aorta as well as aortic wall thickening including in the abdominal aorta

**Fig. 3:**
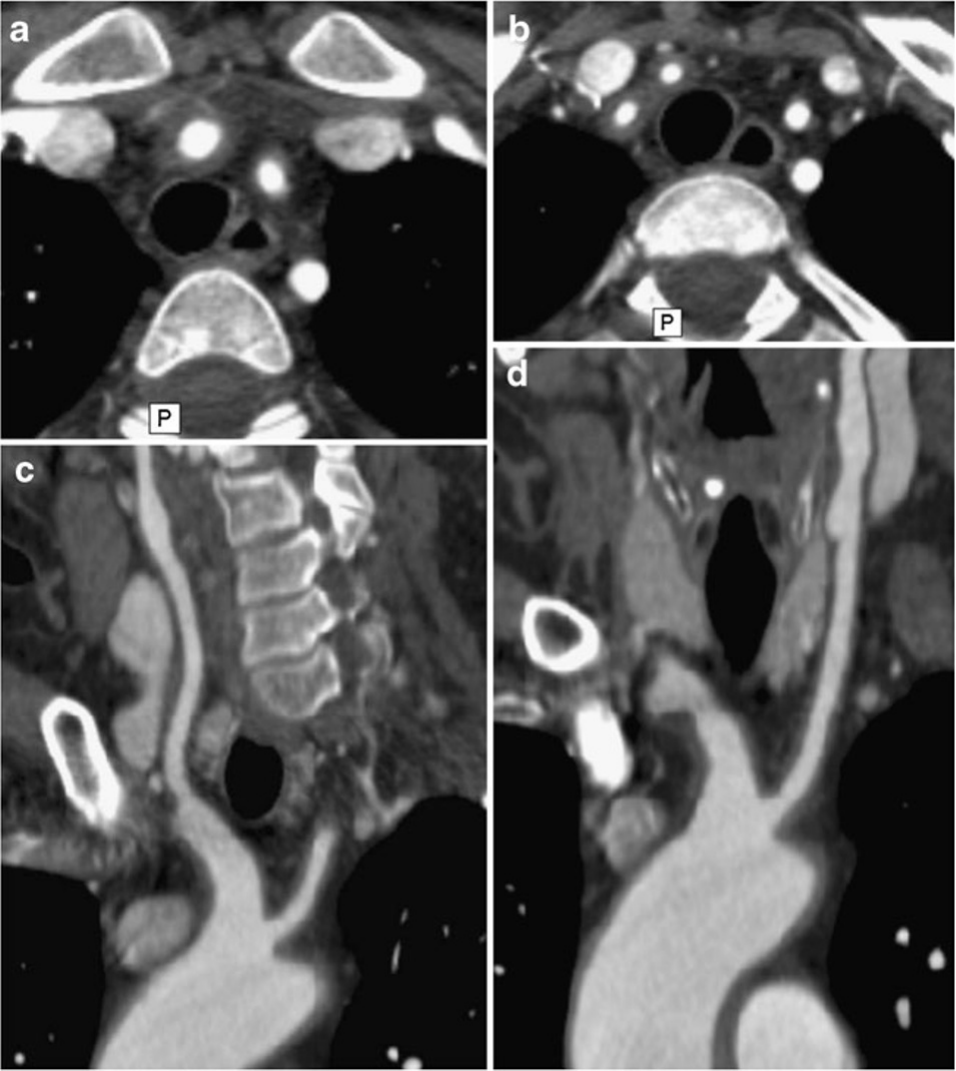
**a**–**d** A 48-year-old woman with weight loss, malaise and headaches. Axial (**a**, **b**) CTA images demonstrate concentric thickening of the brachiocephalic artery, continuing toward its branches: right common carotid artery, right subclavian artery and left subclavian artery (originating from the brachiocephalic artery in this case). Multiplanar curved reformats (**c**, **d**) demonstrate both wall thickening and aneurysmal dilatations along the course of both common carotid arteries (**c***right*, **d***left*) and brachiocephalic artery trunk (**c**). Courtesy of Dr. Ludmila Guralnik, Rambam Health Care Campus, Haifa, Israel

1. Computer tomography
  Acute stage:
  The vast majority of TA cases are diagnosed with CT angiography (CTA) since CTA can depict early findings in the vessel wall and lumen, such as circumferential vessel wall thickening, thrombosis, stenosis, occlusion, vessel ectasia, aneurysms and ulcers (Figs. 1, 2, 3, and 4) [19, 24]. The “double ring” appearance of the thickened aortic wall at contrast-enhanced CT is an early stage finding with a poorly enhanced internal ring of swollen intima and an enhancing outer ring of the inflamed media and adventitia [5, 24].

**Fig. 4:**
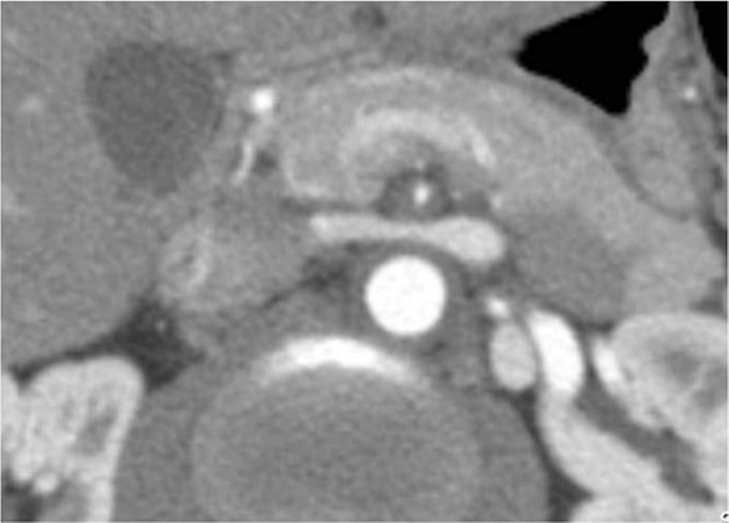
A 36-year-old male with abdominal pain and weight loss over the last 3 months. MDCTA of the abdomen demonstrates diffuse thickening of the aorta and superior mesenteric artery (SMA) with substantial narrowing of the SMA lumen (*arrow*). Courtesy of Dr. Ludmila Guralnik, Rambam Health Care Campus, Haifa, Israel
  That milder degrees of inflammation or wall oedema may not be apparent with CTA is of note. It is considered less sensitive than other modalities such as MR or PET-CT for evaluating the degree of inflammation in the aortic wall [25].
  Since CTA is performed using iodinated contrast, it offers the additional advantage of allowing rapid exclusion of aortic pathologies that may clinically mimic acute aortitis, including aortic dissection, intramural haematoma and penetrating atherosclerotic ulcer.
  Chronic stage:
  Chronic findings include long-standing, burned out aortitis such as linear arterial wall calcification that can be seen after a minimum of 5 years of inflammatory involvement in the aorta or any of the involved vessels, except the ascending aorta [5, 24]. CT can also be used to assess the progression of a potential thoracic aortic aneurysm [1]. High specificity and sensitivity of CT angiography (95 % and 100 %, respectively) [26] as well as high availability make it the imaging modality of choice for the evaluation of TA.
  Although ionising radiation is of concern, new CT low-dose techniques focussing on decreased kilovoltage and novel reconstruction algorithms provide substantial dose reduction and should be considered, especially when patients are young [27].
2. Magnetic resonance imaging
  A major strength of MR imaging is its ability to depict wall abnormalities before luminal changes occur [28]. Gadolinium-enhanced fat-suppressed T1-weighted images are preferred to assess thickening and enhancing of the arterial wall and T2-weighted images for showing high signal of the vessel wall representing mural oedema (Figs. 5 and 6). MR angiography may show stenosis at multiple levels, mural thrombi, thickening of aortic valve cusps and pericardial effusions [18, 28–30]. Signal alterations within the pericardial effusion, reflecting fluid and granulation tissue, can be seen as well [18]. Cine MRI can detect cardiovascular and haemodynamic changes, such as aortic regurgitation in patients with TA [31]; MR angiography demonstrates the anatomical location, degree, extent of stenosis and vascular dilation, and patency of collateral vessels and surgical bypass grafts.

**Fig. 5:**
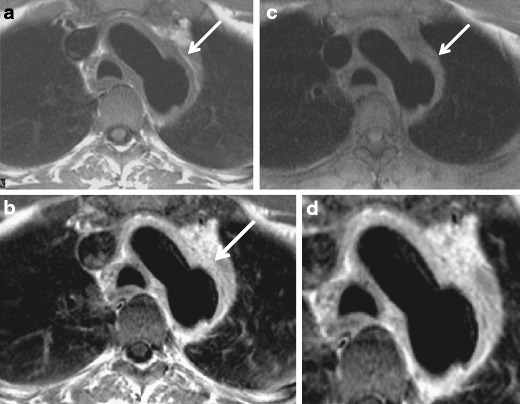
**a**–**d** A 51-year-old male with chest pain. Spin-echo T1-weighted (**a**), post-gadolinium enhanced (**b**) and proton density (**c**) images of the aortic arch show diffuse and relatively uniform thickening of the aortic arch with focal aneurysmal dilatation of the posterior aspect of the distal arch (*arrow*). Vivid gadolinium enhancement is uniform and diffuse (**b**), being even more vivid at the mid-outer portion of the aortic wall (**d**), corresponding to a “double ring” appearance

**Fig. 6:**
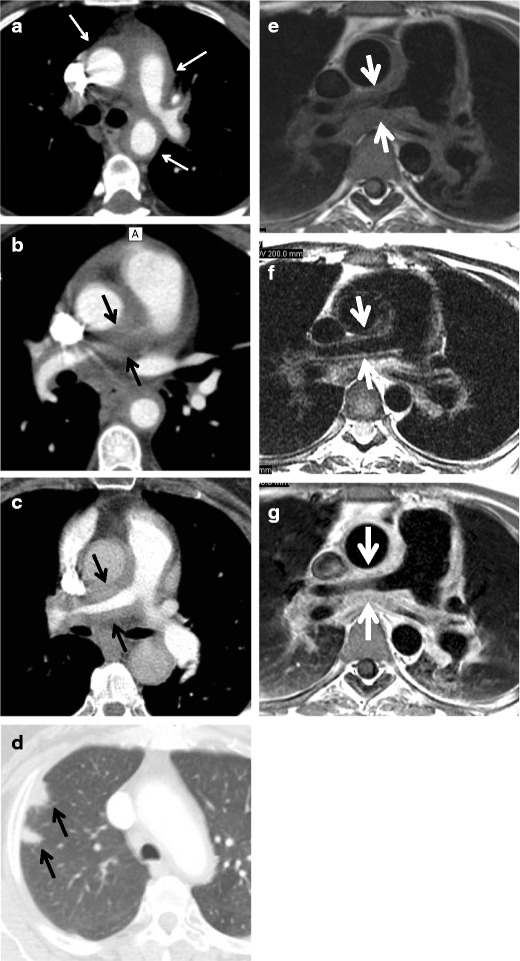
**a**–**g** A 63-year-old man with a prolonged history of Takayasu arteritis. MDCTA axial images of the chest at the level of the main and left pulmonary artery (**a**), right pulmonary artery (**b**, **c**) and *right upper* lobe demonstrate substantial thickening of the pulmonary arterial wall, especially pronounced at the level of the right pulmonary artery with almost complete occlusion of the *right upper* lobe branch and pulmonary infarcts (**d**) (*arrows*). T1-weighted (**e**), T2-weighted (**f**) and GD-enhanced (**g**) MRI images emphasise the same findings as well as substantial wall oedema seen as bright signal on T2-weighted images (**f**) (*arrows*)
  Both contrast-enhanced MRI and CTA play an important role in the early diagnosis of TA, i.e., by demonstrating crescentic or ring-like aortic thickening of more than 3 mm, as well as for assessing disease activity [32].
  Limitations of MR angiography include the possibility that vascular branch points may be improperly interpreted as occlusions (breath-hold techniques have lessened this problem) and that maximum-intensity projection images may falsely accentuate the degree of vascular stenosis [5]. It is therefore advisable to assess the degree of vascular stenoses from the MR angiography source images [18]. Additional drawbacks of MRI are the relatively decreased sensitivity in the assessment of small vessels and poor visualisation of vascular calcifications, as well as the limited anatomical range that can be scanned as opposed to CT. Finally, MRI is commonly less available in the geographic regions where TA is most prevalent [18].
3. Nuclear imaging
  PET-CT may help to identify vasculitis in patients referred for whole-body imaging for constitutional symptoms and fever of unknown origin, as well as to monitor treatment response [11]. The circumferential region of increased metabolic activity in the vessel wall is characteristic of the active phase of TA (Fig. 7). FDG-PET has a reported sensitivity of up to 92 % and a specificity of 89–100 % for the detection of large vessel vasculitis among untreated patients with elevated serum markers [33]. Recently, the use of 18-fluorodeoxyglucose (18F-FDG) PET, either alone or in combination with contrast-enhanced CTA or MRA, has emerged as a potential tool for the initial diagnosis and assessment of disease activity of aortitis caused by Takayasu arteritis with a variable sensitivity of 60–90 % and specificity of 88–100 % [24, 13, 12, 34]. PET-CT may also be useful for monitoring treatment response, reflected in decreases in vessel wall metabolic activity [35]. However, the main limitations of these studies are the small sample size, heterogeneous patient population and inconsistent reference standards [36].

**Fig. 7:**
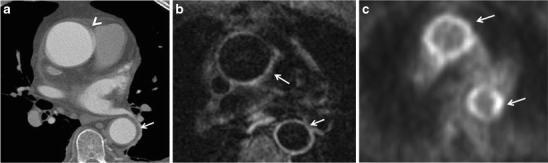
**a**–**c** A 59-year-old woman with low-grade fever, malaise and high white blood cell count. Axial (**a**) CTA image demonstrates normal thickness (*arrowhead*) of the ascending aortic wall with minimal thickening of the descending aortic wall (*arrow*). Axial contrast-enhanced MR image (**b**) shows contrast in the ascending and descending aortic wall corresponding to vivid 18-FDG uptake on PET-CT image (**c**)
  Hybrid imaging with 18F-FDG PET and either CTA or MRA allows more precise anatomic localisation of disease activity with increased uptake of 18F-FDG thought to be a surrogate marker of increased activity of inflammatory cells. The presence of wall thickening, arterial stenosis, luminal thrombus and aneurysm cannot be assessed by PET alone; CTA and MRA are complementary to PET for complete evaluation of the patient with aortitis [24].

#### Complications and therapy

To prevent the known complications of TA such as stenosis, aneurysm formation or occlusion, early treatment with corticosteroids is indicated to suppress inflammatory response. The usual regimen includes high-dose oral steroids (40–60 mg daily), usually for as long as 1–2 years. Unfortunately, up to 50 % of patients relapse during tapering and require additional immunosuppression. Serum markers have limited value in follow-up since disease progression has been shown in the presence of normal serum marker levels [17].

Revascularisation in cases of aortic stenosis or aneurysm is performed when there is secondary vascular organ insufficiency or risk of rupture. Usually the intervention is done in chronic cases after the acute phase inflammation has subsided. Currently, there are no randomised trials of percutaneous or surgical intervention [24].

### Giant cell arteritis

Giant cell arteritis (GCA), also known as temporal arteritis, is an elastic vessel systemic granulomatous vasculitis affecting the aorta as well as its secondary and tertiary branches (large and medium-sized vessels) and usually involves superficial cranial arteries. Aortic involvement occurs in 15 % of GCA patients [37]. As opposed to predominately young patients affected by TA, GCA is usually seen in patients over 50 years old with an incidence peaking in the 8th decade of life [38]. Female predilection is less frequent (3:2 female:male ratio). A higher than usual incidence of disease is seen in Northern Europe (Scandinavia in particular), with lesser frequency in Southern Europe, making genetic predisposition in certain populations likely [1]. Overall, GCA is the most common form of aortitis in North America, accounting for more than 75 % of cases [37]. A strong relationship between GGA and polymyalgia rheumatica has been shown.

#### Clinical presentation

GCA acute and chronic stage pathophysiology is similar to TA [5]. During the acute stage constitutional symptoms such as weight loss, night sweats, malaise and fever are extremely common, affecting half of the patients. In up to 90 % of patients with biopsy-proven disease, scalp tenderness is present. Cranial symptoms (tenderness, headache), jaw claudication, visual changes and neurologic changes are all commonly seen [1].

Vascular inflammation most commonly involves external carotid branches, especially the superior temporal artery and the vertebral arteries. Extracranial arteries are involved in 25 % of cases such as the aorta, coronary arteries and mesenteric arteries [39]. Five-year survival can be affected in cases of widespread disease [40]. Aortic stenosis is less common than in TA [41], although annuloaortic ectasia or ascending aortic aneurysms that can extend into the aortic arch are more common than in TA [42–44]. Thoracic aortic aneurysms are usually seen in the late stages of the disease.

#### Diagnosis

GCA is diagnosed based on the 1990 American College of Rheumatology criteria where three out of five criteria are required for diagnosis, with sensitivity of 94 % and specificity of 91 % [1, 38].

- Age older than 50 years
- Recent-onset localised headache
- Temporal artery pulse attenuation or tenderness
- Erythrocyte sedimentation rate > 50 mm/h
- Arterial biopsy: necrotising vasculitis
- No imaging findings are required for diagnosis

#### Imaging findings

##### CT, MRI, PET-CT and ultrasound

Although no imaging findings are required for diagnosis, imaging might be helpful in diagnosing GCA. Long segment involvement with significant wall thickening and smooth tapering proximal and distal to the lesion on CT and MR are classic radiological findings. The most frequently affected arteries are the subclavian, axillary, femoral, popliteal, tibial and peroneal, with rare involvement of coronary arteries [45]. CT and in particular MR angiography are able to demonstrate vessel wall oedema, which reflects disease activity [46]. CT angiography can reveal luminal changes similar to TA such as stenosis, occlusion, dilatation, aneurysm formation, calcification and mural thrombi [47].

An association between history of GCA and the development of aortic aneurysm has been described, particularly thoracic aortic aneurysm [41]. The frequency of aortic involvement in GCA is unknown, but it has been suggested that all patients with temporal GCA and symptoms of extracranial vascular involvement undergo an imaging study to evaluate the aorta and large thoracic vessels [48].

FDG PET has been shown to be sensitive for extracranial vasculitis but not for intracranial vasculitis on account of its poor spatial resolution [49]. FDG PET reveals abnormal uptake in the aortic arch or large thoracic arteries in more than half of affected patients (sensitivity 56 %, specificity 98 %, positive predictive value 93 %, negative predictive value 80 %) [11, 50].

Ultrasonography is useful in assessing cranial vessels, showing an increased diffuse, circumferential intima-media complex (IMC) thickness in transverse sections (dubbed the ‘macaroni sign’), reflecting inflammatory oedema, increased vascularity or both. In contrast, atherosclerotic lesions are usually characterised by a localised thick IMC pattern [7].

#### Complications and therapy

Aortic aneurysm complicated by acute dissection or aortic valve insufficiency is associated with decreased survival (on average 1.1 years) [41]. The standard therapy, high-dose oral steroids (40–60 mg daily) for 1–2 years, results in rapid improvement but a high relapse rate. Unlike TA, additional immunosuppressive therapy does not affect the course of the disease, but the approach to revascularisation is similar to TA [1, 48].

### Behçet's disease

Behçet's disease is a chronic multisystemic and relapsing inflammatory disorder seen particularly in young males. The clinical triad is recurrent oral and genital ulcers, and uveitis was originally described by the Turkish dermatologist Hulusi Behçet in 1937 [51] . The disease is most frequently seen in the Mediterranean region, Middle East and Far East. The highest prevalence (80–370 per 100,000) was reported in Turkey [52] but it is now increasing in other parts of the world, especially Europe and America, because of migration. A significant association between HLA-B51 and the risk of developing Behçet’s disease supports a genetic predisposition to the disease [53].

#### Diagnosis

The diagnosis is made on the basis of the criteria established by the International Study Group for Behçet’s Disease. The diagnostic criteria require the presence of oral ulceration and at least two of the following: recurrent genital ulceration, eye lesions (anterior and posterior uveitis and retinal vasculitis), skin lesions (erythema nodosum, pseudofolliculitis, papulopustular lesions and acneiform nodules) or a positive pathergy test (development of an erythematous papule or pustule 48 h after a needle prick to the skin) [54].

#### Imaging fındings

**Fig. 8:**
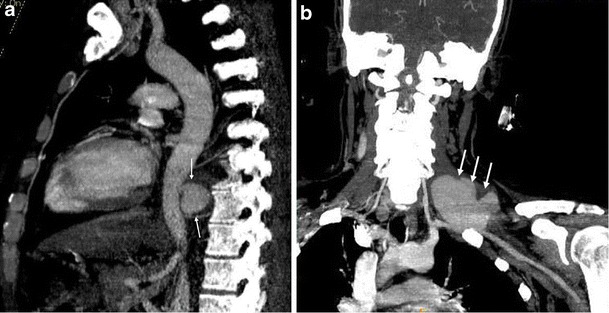
**a**, **b** A 40-year-old male with Behçet's disease. Sagittal maximum-intensity-projection CT image (**a**) shows a saccular aneurysm arising from the distal thoracic aorta (*arrows*). Coronal maximum-intensity-projection CT image (**b**) shows saccular aneurysm arising from the left subclavian artery (*white arrows*)

**Fig. 9:**
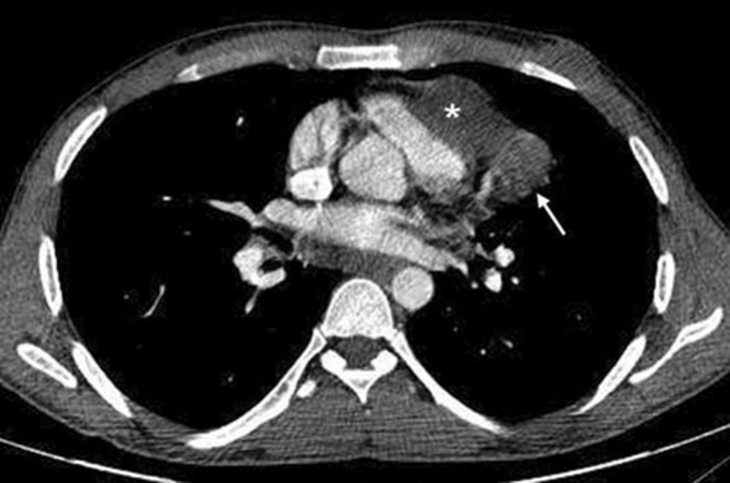
A 24-year-old male with Behçet's disease. Axial CT image shows ruptured pseudoaneurysm of the left anterior descending coronary artery (*white arrow*) with accompanying haematoma (*white asterisk*)

**Fig. 10:**
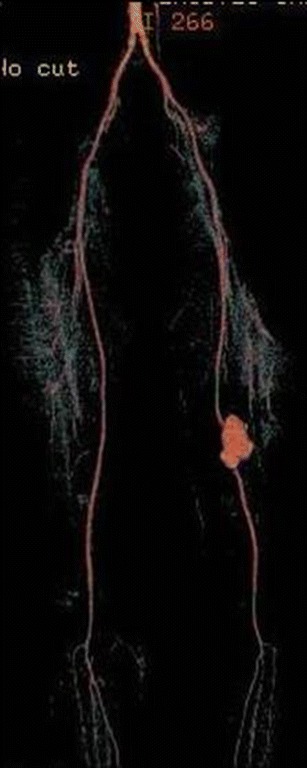
A 34-year-old male with Behçet's disease. 3D colour-coded volume-rendering CT image shows pseudoaneurysm of the superficial femoral artery

Multidetector CT angiography (MDCTA) plays a critical role in the noninvasive assessment of various types and sites of vascular involvement and associated parencyhmal and pleural changes, as well as preoperative treatment planning, because of its multiplanar and vascular reconstruction capabilities (Figs. 7, 8, 9 and 10) [55]. The frequency of vascular involvement in Behçet’s disease is only 25–30 %, but it is the most common cause of mortality [56, 57]. Histopathologically, leukocytoclastic vasculitis is seen in Behçet’s disease. The vasculitic process results in inflammation that leads to occlusion, thrombus and aneurysm formation. The vasculitis can involve various sized arteries and veins (large, medium and small) of the systemic and pulmonary circulation [52, 58]. The small vessel involvement affects the skin, joints, central nervous system and gastrointestinal tract and may result in nonvascular complaints such as erythema nodosum, arthritis, demyelination and gastrointestinal involvement with diarrhoea, gastrointestinal bleeding or perforation [59, 60].

**Fig. 11:**
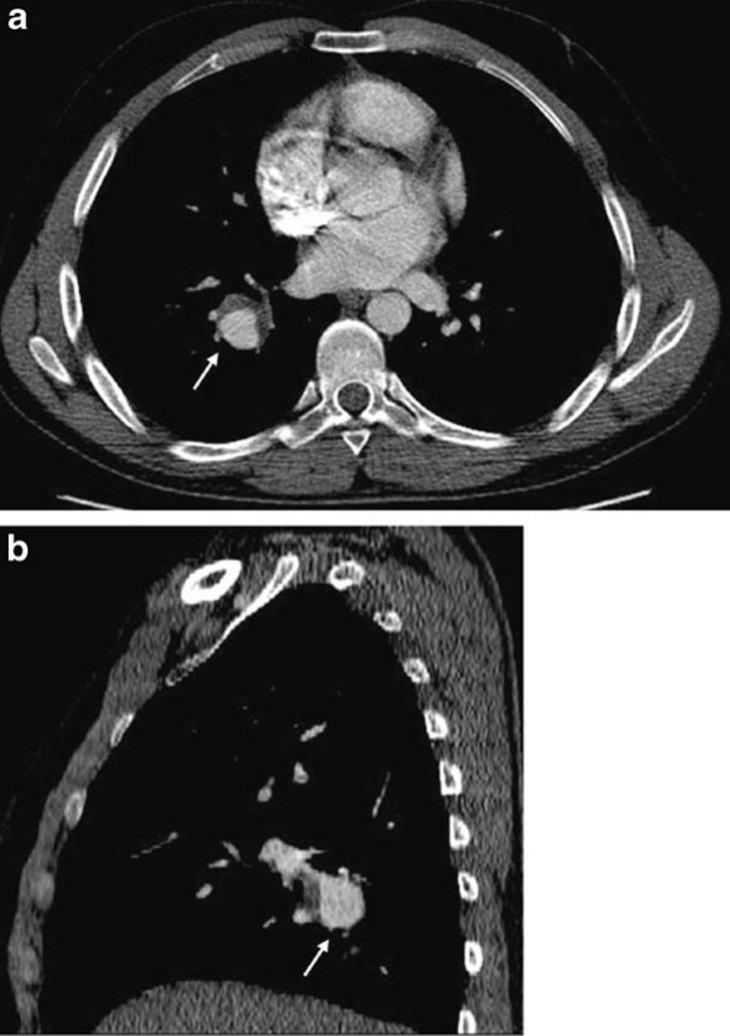
**a**, **b** A 23-year-old male with Behçet's disease. Axial (**a**), sagittal reformatted (**b**) contrast-enhanced CT images show partially thrombosed right pulmonary artery aneurysm (*arrow*)

While arterial involvement is rare, the artery most often affected is the aorta followed by the pulmonary arteries. Although rare, aortic arch branches such as coronary arteries and extremity arteries can also be involved. Aneurysm formation occurs more commonly than occlusion. Histopatologically, inflammation of the vasa vasorum that leads to destruction of the elastic fibres of the tunica media causing aneurysm, pseudoaneurysm formation and rupture is seen [61]. Behçet's disease is the most common cause of pulmonary artery aneurysm (Fig. 11) [62]. Patients with pulmonary arterial aneurysms have a poor prognosis. Thrombosis of the pulmonary arteries is commonly in situ. Although deep vein thrombosis is common, the risk of developing pulmonary emboli is very low because the thrombi are strongly attached to the vasculitic veins. [55].

**Fig. 12:**
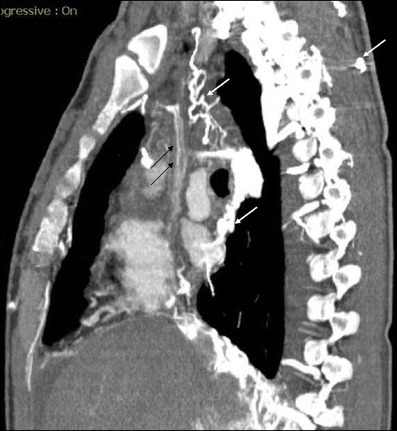
A 20-year-old male with Behçet's disease. Sagittal reformatted CT image shows superior vena cava thrombosis (*black arrows*) and collateral vessels in the mediastinum and chest wall (*white arrows*)

**Fig. 13:**
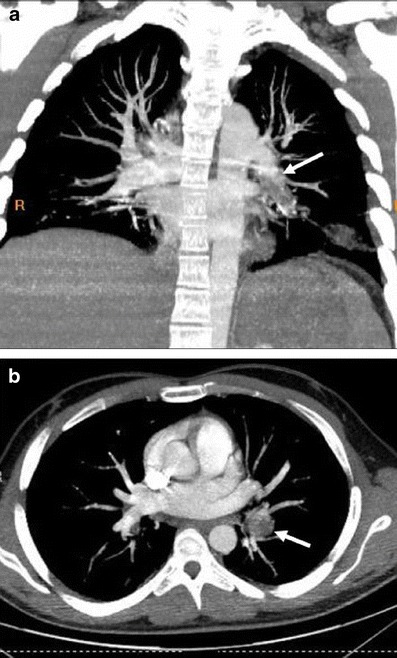
A 31-year-old male with Behçet's disease. Coronal maximum-intensity projection CT image (**a**) shows thrombosed left lower lobe pulmonary artery aneurysm (*arrow*) and pleural-based, wedge-shaped infarction (*arrowhead*). Axial CT image (**b**) also shows a thrombosed left lower lobe pulmonary arterial aneurysm (*arrow*)

In Behçet's disease venous manifestations are more common than arterial involvement. Superficial venous thrombophlebitis is the most frequent venous involvement. Deep vein thrombosis affects the veins of the lower extremities most commonly, followed by superior vena cava, inferior vena cava and upper extremity involvement. Cerebral venous thrombosis may also be seen in Behçet's disease [59]. Venous aneurysms and varices are rarely seen in Behçet disease [63] but superior vena cava (SVC) thrombosis is well known. Thrombosis of adjacent large veins such as the brachiocephalic, subclavian and axillary veins may accompany SVC thrombosis. Collateral veins are seen in chronic stage SVC thrombosis in the neck, mediastinum and chest wall (Figs. 12 and 13) [64]. SVC syndrome due to vasculitis that results in thickening of the vessel wall without evidence of thrombosis may also be seen.

The main cause of sudden death in Behçet's disease is rupture of a large aortic or arterial aneurysm.

#### Hughes-Stovin syndrome

Hughes-Stovin syndrome is characterised by pulmonary arterial aneurysms and systemic venous thrombosis. Hughes-Stovin syndrome is also known as “incomplete Behçet's disease” because of similarities between the vascular manifestations of Behçet's disease and Hughes-Stovin syndrome [59, 65].

#### Complications and therapy

A combination of corticosteroids and cytotoxic agents is used for patients with arteritis. Anticoagulant therapy for deep venous thrombosis should be given carefully in patients with pulmonary arterial aneurysms because of the risk of potentially fatal haemoptysis. Surgical treatment may be considered for large vessel disease refractory to medical treatment [66–68]. Anastomotic pseudoaneurysms and recurrence often occur in surgical repair. Endovascular intervention with a stent graft is an effective alternative to surgery [69].

### Ankylosing spondylitis

Ankylosing spondylitis is part of group of diseases called “spondyloarthropathies” that have a strong association between major histocompatibility complex *HLA B-27* and the absence of rheumatoid factor. Common features of the disease are sacroilitis, inflammatory arthritis or enthesitis, and association with inflammatory bowel disease or psoriasis. This is the first rheumatic disease found to be associated with aortitis, seen in up to 80 % of patients with AS, in particular aortic root involvement and aortic valve disease [70]. More than half of the patients demonstrate aortic wall thickening [71]. Since aortic valve involvement is associated with valvular insufficiency, it can lead to heart failure and death. Heart block has been described as part of the disease because of extension of the subaortic fibrotic process into the interventricular septum causing conduction abnormalities (second common cardiovascular manifestation occurring in AS patients) [71]. The frequency of aortic insufficiency and regurgitation parallels that of the duration of the disease [71].

#### Clinical presentation

Ankylosing spondylitis, the most common variant of spondyloarthropathies, often begins with back pain and stiffness during the second or third decade of life. Contrary to TA and GCA, it affects men two to three times as often as women. Disease usually worsens with inactivity. Constitutional symptoms (malaise or fever) as well as acute anterior uveitis (up to 40 % of patients) are common features [71]

#### Diagnosis

Diagnosis of AS requires four of the five criteria.

- Onset of pain at age younger than 40 years
- Back pain for longer than 3 months
- Morning stiffness
- Subtle symptom onset and
- Improvement with exercise
- No imaging findings are required for diagnosis

#### Imaging findings

Because the aortic root and aortic valve are most commonly affected, all three modalities, echocardiography, MR imaging and CT angiography, are useful for diagnosis. Nodular appearance of the aortic valve and presence of aortic regurgitation are better seen with echocardiography. Involvement of coronary arteries is rare but can be seen with CT coronary angiography. Imaging is also useful for pre-surgical planning in cases of aortic root expansion or aortic valvular malfunction [70, 71].

### Aortitis in association with other rheumatologic disorders

Additional rheumatic diseases associated with aortitis are: relapsing polychondritis (RP), rheumatoid arthritis (RA), systemic lupus erythematosus and Cogan's syndrome, with RP and RA being relatively more frequently described.

#### Relapsing polychondritis

Relapsing polychondritis can affect the elastic elements of the cardiac valves and aorta as part of the multisystem inflammatory autoimmune disorders represented by aortic root dilatation and aortitis. The prevalence of cardiac involvement is 15–45 % and includes aortic dilatation, with secondary regurgitation, mitral regurgitation and aortitis caused by cystic degeneration of collagen, destruction of elastic fibres, lymphocytic infiltration and decreased acid mucopolysaccharide content [72].

The acute and chronic phases of relapsing polychondritis are similar to those seen in TA and GCA, although the entire aortic wall is involved as opposed to primarily the media and adventitia [73]. Aortic wall calcification and ossification with nodular wall formation have also been described [74] as well as aneurysm formation in the thoracic and abdominal aorta (5 % of cases) and vasculitis obliterans in medium-sized and large arteries [75, 76].

#### Rheumatoid artritis

Aortitis is rare in RA, which is seen in about 5 % of cases with even less frequent aneurysm formation [77]. The aortic valve and annulus may also be affected by granulomatous or nongranulomatous inflammation, leaflet thickening and secondary regurgitation [78]. Involvement of the coronary ostia, if present, may lead to myocardial ischaemia [15]. Life-long therapy with corticosteroids carries the risk of spontaneous rupture of pre-existing, possibly multiple, aortic aneurysms [79, 80].

#### Idiopathic isolated aortitis

**Fig. 14:**
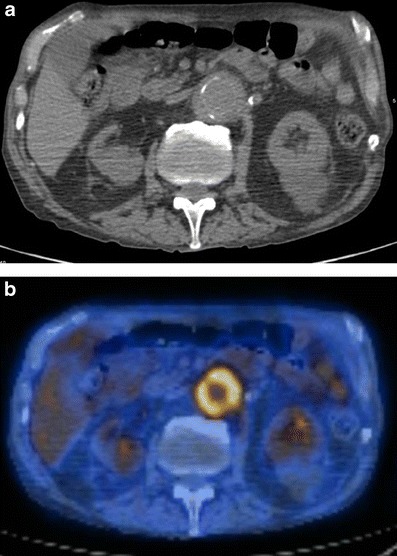
A 75-year-old male with abdominal pain. An axial non-contrast-enhanced CT image (**a**) of the abdomen demonstrates circumferential homogeneous thickening of the aortic wall up to 5 mm corresponding to vivid circumferential 18-FDG uptake on PET-CT image (**b**). Subsequent biopsy of the aortic wall was consistent with idiopathic inflammation

This is a rare form of aortitis complicated by a lack of any known systemic disease. It may present with symptoms related to aortic inflammation such as back pain, abdominal pain and/or elevated inflammatory markers, and both the thoracic and abdominal aorta can be involved. Incidental diagnosis is also possible [24]. Usually silent, it is complicated by the formation of aneurysms in the thoracic aorta or abdominal aorta, where up to 10 % of all aortic aneurysms might be attributed to inflammatory aortitis. A combination of thoracic and abdominal aneurysms is also possible [24]. Idiopathic inflammatory aneurysms differ from atherosclerotic aneurysms because of the presence of dense perianeurysmal fibrosis and a thickened aortic wall [81] (Fig. 14). Acute renal failure caused by ureterine obstruction has been reported in patients with retroperitoneal fibrosis, part of the spectrum of idiopathic isolated aortitis [82] The male:female ratio is 2:3. Studies have shown that new aneurysms can develop in 25 % of patients who did not receive anti-inflammatory therapy [83].

## Infectious aortitis

**Fig. 15:**
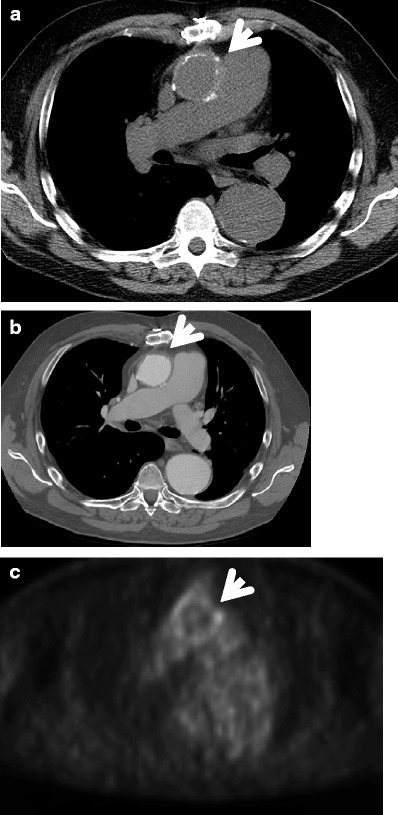
A 67-year-old female several months after ascending aneurysm repair, with low-grade fever and malaise. Axial non-contrast-enhanced (**a**) and contrast-enhanced (**b**) images of the chest demonstrate an ascending aortic graft with surrounding minimal aortic wall thickening (*arrowheads* in **a** and **b** respectively). The area was shown to be positive for moderate 18-FDG uptake on the PET-CT image (*arrowhead* in **c**). Blood cultures revealed *Staphylococcus aureus* infection, concerning for infectious aortitis

The aorta is normally very resistant to infection; however, an abnormal aortic wall, like that associated with atherosclerotic disease, preexisting aneurysm, cystic medial necrosis, diabetes, vascular malformation, medical devices or surgery, makes it more susceptible to infection. The aetiologies vary. In the antibiotic era the most frequent causative agents are *Staphylococcus aureus, Salmonella, Pneumococcus*, *Escherichia coli, Treponema pallidum and* other *Treponema* species (10–25 years after the initial spirochetal infection), *Candida, Aspergillus and Tuberculosis* [5] (Fig. 15).

The spread of disease is usually contiguous, spread from adjacent thoracic structures (mediastinitis, abscess, infected lymph nodes, infectious pericarditis, empyema, paravertebral abscess), although septic emboli from underlying bacterial endocarditis or haematogenous dissemination of bacteria in the setting of sepsis or intravenous drug abuse are not uncommon [5, 84].

An ascending thoracic aorta, aortic arch and descending thoracic aorta can all be affected, as well as prosthetic aortic grafts and aortic homografts. Typically, sites of infected aneurysms are opposite the great vessels in the aortic arch or opposite the visceral arteries in the abdomen. Saccular aneurysms are most common, but infected aneurysms can be fusiform and often even pseudoaneurysms [5].

### Imaging findings

Contrast-enhanced CT is the imaging modality of choice, demonstrating aortic wall thickening, periaortic fluid or soft-tissue accumulation, rapidly progressing saccular aneurysm or pseudoaneurysm, and occasionally air in the aortic wall [85].

#### Pyogenic infection

This event usually affects native aorta secondary to bacteremia with endovascular seeding. If left untreated, it may progress to mycotic aneurysm [5]. Pneumococcal aortitis is seen mainly in the elderly secondary to bacteremia as well [86].

#### Tuberculous aortitis

This disorder is exceedingly rare and may mimic Takayasu aortitis. To avoid delayed diagnosis or unnecessary immunosuppressive therapy, this diagnosis should be considered in patients with aortitis or atypical aortic aneurysm who have a history of pulmonary or extrapulmonary tuberculosis or who present with a cavitary lung lesion, pleural effusions or lymphadenitis [24].

Usually the distal aortic arch and descending aorta are involved, resulting from either direct extension from the thoracic spine involved with tuberculous osteomyelitis, mediastinal lymph nodes, empyema or pericarditis, or from haematogenous spread [5]. The association between vertebral tuberculosis and perforation is high, particularly with the presence of a cold abscess [87].

#### Syphilitic aortitis

Though quite rare today, sexually transmitted diseases can present as syphilitic aortitis, syphilitic aortic aneurysm, syphilitic aortic valvulitis with aortic regurgitation or syphilitic coronary ostial stenosis. Chronic aortic inflammation results in fibrosis and wrinkling of the intima with subsequent aneurysm formation. Calcification of the ascending aorta is possible but uncommon. The ascending thoracic aorta is most commonly involved (60 %), followed by the aortic arch (30 %) [88].

## Summary

Aortitis comprises a group of rare but important diseases, the most common of which are Takayasu arteritis, giant cell arteritis and Behçet's disease, predominantly seen in specific areas, migration notwithstanding. Imaging is rarely used for primary diagnosis but is important for the overall assessment of the patient and for follow-up. The primary imaging method is contrast-enhanced CT where these diseases classically show abnormalities of wall thickness, vascular diameter and wall density. Additional imaging can be performed with MRI, and recently PET CT has been shown to be a helpful adjunct. Radiologists should be familiar with the most common manifestations of aortitis because imaging can be critical in the initiation of appropriate management and therapy.
